# Calaspargase-Pegol-Mknl Combined with BCL-2 and MCL-1 Inhibition for Acute Myeloid Leukemia

**DOI:** 10.3390/ijms252313091

**Published:** 2024-12-05

**Authors:** Dominique Bollino, Xinrong Ma, Kayla M. Tighe, Andrea Casildo, Katharina Richard, Antonino Passaniti, Brandon Carter-Cooper, Erin T. Strovel, Ashkan Emadi

**Affiliations:** 1 Department of Medical Oncology, Cancer Institute, West Virginia University, Morgantown, WV 26506, USA; 2 Department of Medical Oncology, School of Medicine, West Virginia University, Morgantown, WV 26506, USA; 3 The Biology Department, University of Maryland Marlene and Stewart Greenebaum Comprehensive Cancer Center, Baltimore, MD 21201, USA; 4 Department of Microbiology and Immunology, University of Maryland School of Medicine, Baltimore, MD 21201, USA; 5 Department of Pathology, University of Maryland School of Medicine, Baltimore, MD 21201, USA; 6 Department of Pharmacology, University of Maryland School of Medicine, Baltimore, MD 21201, USA

**Keywords:** acute myeloid leukemia, BCL-2, MCL-1, asparaginase

## Abstract

Our previous studies have demonstrated that pegcrisantaspase (PegC), a long-acting *Erwinia* asparaginase, synergizes with the BCL-2 inhibitor Venetoclax (Ven) in vitro and in vivo; however, the anti-leukemic activity of *E. coli*-derived asparaginases in combination with BCL-2 inhibition, and potential synergy with inhibitors of MCL-1, a key resistance factor of BCL-2 inhibition, has yet to be determined. Using a combination of human AML cells lines, primary samples, and in vivo xenograft mouse models, we established the anti-leukemic activity of the BCL-2 inhibitor S55746 and the MCL-1 inhibitor S63845, alone and in combination with the long-acting *E. coli* asparaginase calaspargase pegol-mknl (CalPegA). We report that CalPegA enhances the anti-leukemic effect of S55746 but does not impact the activity of S63845. The S55746-CalPegA combination inhibited protein synthesis and increased eIF4E/4EBP1 interaction, suggesting an inhibition of translational complex formation. These results support the clinical evaluation of CalPegA in combination with BCL-2 inhibition for AML.

## 1. Introduction

Targeted therapies that address specific molecular alterations, such as overexpression of anti-apoptotic proteins, are reshaping the AML therapeutic landscape. Anti-apoptotic members of the BCL-2 family of proteins, including BCL-2 and BCL-X_L_, are frequently overexpressed in AML. Through binding and sequestering pro-apoptotic proteins, such as BAX and BAK, these proteins inhibit mitochondrial outer membrane permeabilization, thereby preventing the release of cytochrome c and subsequent activation of the caspase cascade. Anti-apoptotic BCL-2 family proteins are therefore promising therapeutic targets in AML. Navitoclax, a BH3 mimetic that inhibits both BCL2 and BCL-X_L_, was the first potent BCL2 inhibitor to enter clinical trials; however, profound thrombocytopenia proved to be the dose-limiting toxicity due to the critical role of BCL-X_L_ in platelet survival [1]. Venetoclax (Ven), a highly selective BCL-2 inhibitor, was FDA-approved in 2018 for treating newly diagnosed AML in elderly patients aged 75 years and up, in combination with DNA methyltransferase inhibitors [2]. S55746, a BCL-2 inhibitor with binding sites distinct from Ven, has also progressed to clinical testing [3,4]. BCL-2 inhibitor use has been associated with resistance mechanisms, including the upregulation of the anti-apoptotic protein MCL-1. Similar to BCL-2, MCL-1 can also bind and sequester pro-apoptotic proteins to prevent apoptosis induction. The identification of therapeutic strategies that can combat BCL-2 inhibitor resistance is highly desirable.

Metabolic reprogramming of cancer cells to fuel growth can introduce targetable vulnerabilities, such as increased demand for amino acids. Asparaginases are enzymes that deplete plasma asparagine and glutamine and can therefore be used therapeutically for cancers that are dependent on these amino acids. Asparaginases are part of well-established multi-agent chemotherapeutic regimens in pediatric and adult patients with acute lymphoblastic leukemia (ALL), and emerging evidence has demonstrated their anti-leukemic activity in AML [5,6,7]. Commercially available asparaginases are isolated from either *E. coli* or *Erwinia chrysanthemi* (also called crisantaspase), the latter having a higher glutaminase activity [8,9]. Our previous studies in AML models have shown that the long-acting pegylated crisantaspase pegcrisantaspase (PegC) synergizes with Ven and the anti-leukemic activity of Ven-PegC is associated with the inhibition of cap-dependent mRNA translation and downregulation of MCL-1 expression (5). Given the role of MCL-1 upregulation in BCL-2 inhibitor resistance and that MCL-1 is essential for the development and growth of AML [10], MCL-1 inhibition using small molecule inhibitors has been under investigation. S63845, a specific MCL-1 inhibitor, has potent in vivo anti-tumor activity in several cancer models, including leukemia [11].

We hypothesized that anti-AML synergism between BCL-2 inhibition and asparaginase products is a pharmacologic class effect independent of any specific agents. To test this hypothesis and to broaden the impact of this metabolically driven novel treatment strategy, we used the FDA-approved long-acting *E. coli*-derived asparaginase calaspargase-pegol-mknl (CalPegA) in combination with the BCL-2 inhibitor S55746 in AML. Additionally, we investigated the combination of CalPegA with the MCL-1 inhibitor, S63845, and hypothesized that due to redundancy in the targeted pathway, this combination would not have an additive or synergistic anti-AML effect. Here, we report the in vitro, in vivo, and mechanistic findings of our experiments testing these hypotheses.

## 2. Results

### 2.1. CalPegA Potentiates the Anti-Proliferative Activity of BCL-2, but Not MCL-1, Inhibition

**Figure 1:**
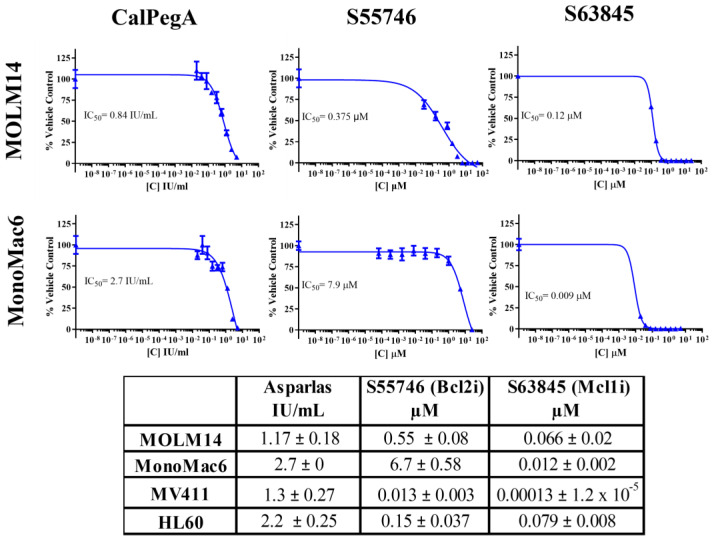
AML cell lines are sensitive to CalPegA, S55746, and S63845. AML cell lines were plated overnight and then treated the next day with serially diluted S55746, S63845, or CalPegA. Cell proliferation was assessed 72 h after treatment using WST-1. Dose–response curves were generated and IC_50_ values were calculated using GraphPad Prism. Representative dose curves are shown for MOLM14 and MonoMac6. The table contains IC_50_ values ± standard error of margin (SEM) for all AML cell lines tested.

Using a panel of four human AML cell lines (MOLM14, MV411, MonoMac6, HL60), we established the single-agent anti-leukemic activity of the BCL-2 inhibitor S55746, the MCL-1 inhibitor S63845, and CalPegA. CalPegA resulted in a dose-dependent inhibition of AML cell proliferation, with IC_50_ values ranging from 1.17 to 2.7 IU/mL. Single-agent S55746 and S63845 treatment inhibited AML cell proliferation with IC_50_ values ranging from 0.01 to 6.7 µM and 0.0001 to 0.08 µM, respectively. Representative dose curves from MOLM14 and MonoMac6 cells and a summary table of IC_50_ for all cell lines tested are shown in Figure 1.

**Figure 2:**
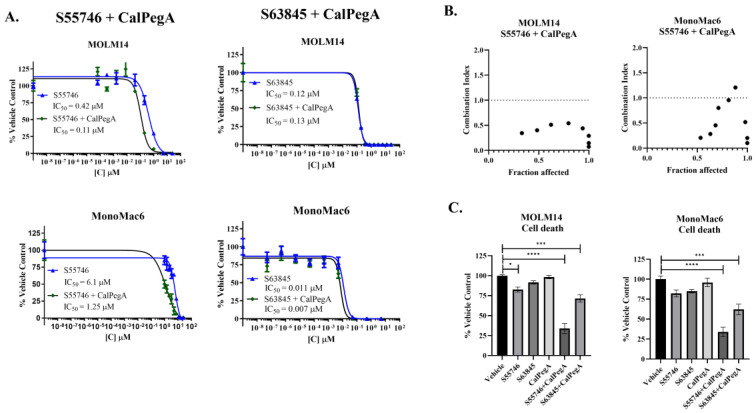
CalPegA potentiates anti-leukemic activity of S55746, but not S63845. (**A**) MOLM14 and MonoMac6 cells were treated with serially diluted S55746 or S63845 alone or in combination with CalPegA (IC_20_). Cell proliferation was measured 72 h after treatment by WST-1. (**B**) MOLM14 and MonoMac6 cells were treated with fixed-ratio doses of CalPegA and S55746 for 72 h followed by WST-1 termination. Combination indexes (CI) were calculated using Compusyn software. (**C**) The percentage of viable cells after treatment with S55746, S63845, or CalPegA alone and in combination at the IC_50_ values for 72 h was determined by trypan blue exclusion. Data are expressed as the percentage of the vehicle control and statistical analyses between the vehicle and treatment groups were performed using unpaired *t*-tests. **** *p* < 0.0001, *** *p* < 0.001, * *p* < 0.05.

To determine if CalPegA can enhance the anti-leukemic effect of BCL-2 or MCL-1 inhibition, MOLM14 and MonoMac6 cells were treated with dose curves of S55746 or S63845 alone or in combination with a fixed dose of CalPegA. In both cell lines, the addition of a small dose (IC_20_) of CalPegA potentiated the anti-leukemic effect of S55746, reducing the IC_50_ approximately 4- to 5-fold. In contrast, the activity of S63845 was not substantially impacted by the addition of CalPegA in either AML cell line (Figure 2A). Next, we investigated whether CalPegA could potentiate the effect of S63845 or S55746 in 4 patient-derived primary AML cells with various cytogenetics and mutational characteristics (Supplemental Figure S1). Potentiation of S55746 by CalPegA was observed in 3 of the 4 primary AML patient samples, with the IC_50_ of S55746 reduced 4.5-, 18-, and 156-fold. Consistent with our cell line data, CalPegA did not enhance S63845 activity (Supplemental Figure S2).

We next used median effect analysis based on the Chou Talalay theorem [12] to determine if the combination of CalPegA and S55746 was synergistic. MOLM14 and MonoMac6 cells were treated with serially diluted CalPegA and S55746 alone and in fixed ratios, and combination index (CI) values were calculated. A CI  <  1 is synergistic, a CI  =  1 is additive, and a CI  >  1 is antagonistic. The CalPegA-S55746 combination resulted in CI values of <1 in all doses tested for MOLM14 and in all except 1 in MonoMac6 cells (Figure 2B), suggesting synergism. When the same experiment was conducted using the combination of CalPegA and S63845, the CI values for most doses tested were not synergistic, further supporting that CalPegA does not enhance the activity of S63845 (Supplementary Figure S3).

### 2.2. CalPegA Enhances Cell Death in S63845 and S55746 Treated AML Cells

To determine whether the monotherapy or combination treatment can result in AML cell death in addition to proliferation suppression, we used trypan blue exclusion to measure the percentage of cell death 72 h after co-exposure of S63845 or S55746 with CalPegA. In MOLM14 cells, single-agent S55746 resulted in a modest (17%) decrease in cell viability, whereas the combination of CalPegA and S55746 decreased cell viability by a mean of 66% compared to vehicle-treated cells. The combination of CalPegA and S63845 decreased cell viability by an average of 28% compared to vehicle-treated cells. Similarly, in MonoMac6 cells, single-agent treatment did not induce cell death but the addition of CalPegA to either S55746 or S63845 resulted in a 66% and 38% decrease in viability, respectively, compared to vehicle-treated cells (Figure 2C). To assess the induction of apoptosis, we performed immunoblotting for total and cleaved caspase 3 and cleaved PARP 24 h after treatment. In MOLM14 cells, both S55746 and S63845 treatment increased cleaved caspase 3 and PARP, and S55746-mediated cleavage was enhanced by the addition of CalPegA. Single-agent S55746 and S63845 resulted in minimal caspase 3 and PARP cleavage in MonoMac6 cells, which was enhanced by the addition of CalPegA (Supplementary Figure S4). These results are consistent with previously reported data indicating limited anti-AML activity of BCL-2 inhibitors when used as monotherapy, underscoring the importance of combination therapy for AML, particularly in the relapsed and refractory settings [13,14,15].

Given that CalPegA did not induce cell death in AML cells, as indicated by trypan blue exclusion assay and the absence of caspase-3 and PARP cleavage in immunoblot analysis, we next sought to assess the cytostatic effects of CalPegA. MOLM14 and MonoMac6 cells were treated with vehicle control, CalPegA, S55746, or S63845, either as single agents or in combination at their respective IC_50_ values. Culture expansion relative to the vehicle control was measured 72 h post-treatment. As shown in Supplementary Figure S5A, CalPegA alone significantly inhibited AML cell culture growth, demonstrated by a reduced fold expansion compared to the vehicle. The other treatment groups also exhibited reduced culture expansion, which can be attributed, at least in part, to the induction of cell death. To further evaluate anti-proliferative effects, cell cycle analysis using propidium iodide staining was performed on MOLM14 and MonoMac6 cells 48 h after treatment with vehicle control, CalPegA, S55746, or S63845, either as single agents or in combination at their respective IC_50_ values. In MOLM14 cells, CalPegA treatment alone and in combination with both S55746 and S63845 resulted in a significant reduction of cells in S phase. In MonoMac6 cells, all treatment groups except S63845 alone resulted in an increase of cells in G0/G1. Additionally, S63845 increased the percentage of cells in S phase, whereas S55746, CalPegA, and S55476 + CalPegA treatment all resulted in a reduced percentage of cells in S phase. The percentage of cells in G2/M were decreased in all treatment groups relative to vehicle control (Supplementary Figure S5B).

### 2.3. The Combination of S55746 and CalPegA Inhibits Global Protein Synthesis and Increases eIF4E/4EPB1 Interaction

**Figure 3:**
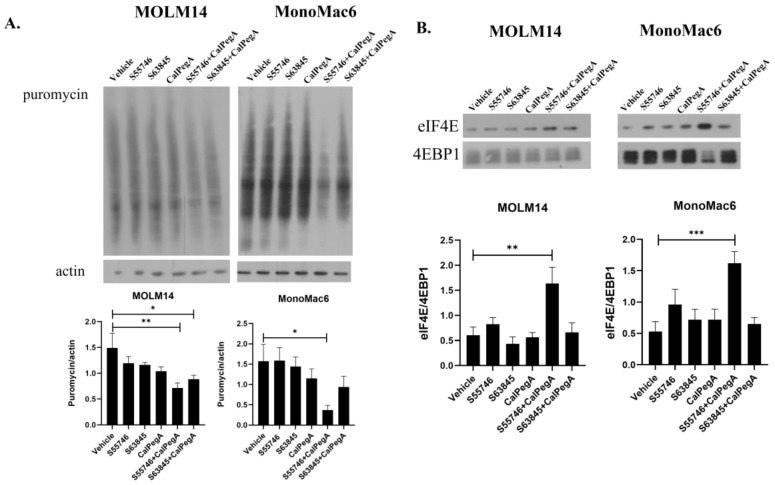
S55746-CalPegA combination inhibits global protein synthesis and increases eIF4E/4EPB1 interaction. (**A**) MOLM14 and MonoMac6 cells were treated with S63845, S55746, and CalPegA alone or in combination (IC_50_ values) for 24 h followed by a 20 min incubation with puromycin (1 µg/mL). Cell lysates were subjected to immunoblotting with the anti-puromycin antibody (SUnSET [surface sensing of translation] assay). Actin was used as a loading control. The bar diagram shows the mean ± SEM for at least three independent experiments, and unpaired *t*-tests were used to compare puromycin intensity in the vehicle group to the treatment groups. ** *p* < 0.01, * *p* < 0.05. (**B**) Protein lysates (250 µg) from MOLM14 and MonoMac6 cells treated for 24 h, as in (**A**), were incubated with antibody against 4EBP1 overnight followed by incubation with Protein A beads. The beads were washed, resuspended in SDS sample loading buffer, and boiled, and then the proteins were separated on 4–15% polyacrylamide gels. Membranes were immunoblotted with antibodies against eIF4E and 4EBP1, and results are expressed as the ratio of eIF4E to 4EBP1. The bar diagram represents densitometric quantification of three independent experiments. *** *p* < 0.001, ** *p* < 0.01, * *p* < 0.05.

Cap-dependent mRNA translation initiates with the binding of eukaryotic translation initiation factor 4E (eIF4E) to 5′ mRNA caps and the subsequent recruitment of the eIF4F initiation complex. When unphosphorylated, the translation repressor protein 4E binding protein 1 (4EBP1) binds eIF4E and inhibits the development of the cap-binding complex. Phosphorylation of 4EBP1 downstream of mTOR pathway activation causes its release from eIF4E to allow cap-dependent translation to proceed [16]. We have previously reported that the combination of Ven with PegC inhibits cap-dependent mRNA translation downstream of mTOR signaling, thereby reducing protein synthesis [5]. To determine if CalPegA in combination with S55746 or S63845 impacts protein synthesis, we assessed global protein synthesis using puromycin incorporation. MOLM14 and MonoMac6 cells were treated with either vehicle or CalPegA, S55746, or S63845 alone or in combination at the IC_50_ values, followed by a short exposure to puromycin 24 h after treatment. Puromycin incorporation into nascent polypeptides was used as a surrogate for global protein synthesis and was detected in whole cell lysates using an anti-puromycin antibody. In both cell lines, we found that puromycin incorporation was significantly diminished by the combination of CalPegA and S55746, indicating decreased protein synthesis (Figure 3A).

To determine the impact of S55746 or S63845 with or without CalPegA on mRNA translation, we measured the association of eIF4E and 4EBP1 to assess translation initiation complex formation. Immunoprecipitation of 4EBP1 revealed that treatment with the S55746-CalPegA combination increased the binding of eIF4E to 4EBP1 compared to single-agent treatments, suggesting an inhibition of cap-dependent mRNA translation (Figure 3B).

### 2.4. In Vivo Tolerability of CalPegA, S55746, and S63845

Prior to in vivo efficacy studies, we first established the tolerability of each agent by treating non-leukemia-bearing NRG mice with increasing doses of each agent. To determine the maximum tolerated dose (MTD) of S55746, NRG mice were treated with 25, 50, and 100 mg/kg of S55746 5 days a week by oral gavage. For S63845, mice were treated intravenously (IV) with 25 mg/kg twice weekly, 50 mg/kg once weekly, and 50 mg/kg twice weekly. The doses for S55746 and S63845 were chosen based on reported doses in the literature [3,11,17]. For CalPegA, mice were injected with CalPegA IV once weekly at 250, 500, and 1000 IU/kg, based on tolerability we previously observed with PegC. Body weight changes relative to the vehicle-treated group were calculated over time to monitor toxicity. S55746 was tolerable at all doses tested and based on our previous work with the combination of Ven and PegC, we chose to move forward with a dose of 75 mg/kg for the efficacy studies (Supplemental Figure S6). S63845 was also tolerable at all doses tested; however, there was weight fluctuation in the 50 mg/kg twice weekly group (Supplementary Figure S6), so we chose a dose of 25 mg/kg twice weekly to ensure tolerability when combined with CalPegA. Unexpectedly, CalPegA was not tolerated at any of the doses tested, as evidenced by the rapid weight loss compared to vehicle controls (Supplemental Figure S6). After consultation with the manufacturer, we decreased the dose to 100 IU/kg for the efficacy studies.

### 2.5. CalPegA Potentiates the Anti-Leukemic Activity of S55746 In Vivo

**Figure 4:**
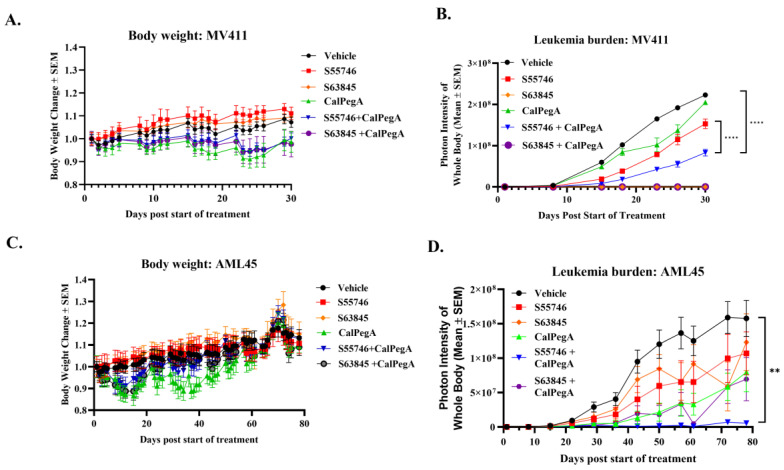
CalPegA potentiates the anti-leukemic activity of S55746 in vivo. (**A**,**B**) MV411-luc cells (1 × 10^6^) were injected IV into NRG mice, and after confirmation of engraftment, mice were treated with either vehicle, CalPegA (100 IU/kg, IV, 1×/week), S55746 (75 mg/kg, oral gavage, 5×/week), S63845 (25 mg/kg, IV, 2×/week), CalPegA + S55746, or CalPegA + S63845. (**A**) Percent body weight changes versus time. (**B**) Photon intensity (leukemia burden) over time. (**C**,**D**) AML45-luc cells (1 × 10^6^) were injected IV into NRG mice, and after confirmation of engraftment, mice were treated as in (**A**). Treatment for all groups was paused from days 9–19 due to weight loss. Treatment resumed for 2 weeks, and then CalPegA dose was reduced to 75 IU/kg after day 36 due to observed weight loss. (**C**) Percent body weight changes versus time. (**D**) Photon intensity (leukemia burden) over time. Statistical analysis was performed using one-way ANOVA. **** *p* < 0.0001, ** *p* < 0.01.

The in vivo efficacy of the S55746/S63845-CalPegA combinations was first evaluated using a luciferase-expressing MV411 orthotopic model. MV411 cells were injected IV into NRG mice and in vivo imaging was used to confirm leukemia engraftment. Mice were treated with either vehicle, CalPegA (100 IU/kg, IV, 1×/week), S55746 (75 mg/kg, oral gavage, 5×/week), S63845 (25 mg/kg, IV, 2×/week), CalPegA + S55746, or CalPegA + S63845. Body weight and tumor burden were measured over time. All groups maintained their body weight, indicating the treatments were tolerable (Figure 4A). The S55746-CalPegA combination reduced tumor burden more effectively than either single agent. S63845 treatment was highly effective at inhibiting leukemia growth, but consistent with our in vitro findings, this effect was not improved by the addition of CalPegA (Figure 4B).

To further evaluate the impact of BCL-2/MCL-1 inhibition in combination with CalPegA on leukemia cell growth in vivo, we utilized a previously established [5] luciferase-expressing patient-derived xenograft (PDX) model from primary cells that were cryopreserved from a patient with relapsed complex karyotype AML (AML45-luc). AML45-luc cells were injected IV into NRG mice and imaged 3 days after injection to confirm engraftment. Mice were treated with either vehicle, CalPegA (100 IU/kg, IV, 1×/week), S55746 (75 mg/kg, oral gavage, 5×/week), S63845 (25 mg/kg, IV, 2×/week), CalPegA + S55746, and CalPegA + S63845 and imaged weekly to monitor AML burden (Figure 4D). Due to weight loss in the CalPegA groups, treatment for all groups was paused from days 9–19 to allow the mice to recover. After resuming treatments for 2 weeks, weight loss was once again observed in the CalPegA-received groups, prompting us to reduce the dose of CalPegA to 75 IU/kg after day 36. No further weight loss was observed (Figure 4C) at the new dosage. The study was terminated at day 78 post-treatment initiation when the disease burden for the vehicle-treated mice was approaching endpoint so that blood and bone marrow could be collected at the same timepoint for pharmacodynamic studies. Compared to vehicle, the S55746-CalPegA combination was the only treatment that significantly inhibited leukemia growth. Unlike the MV411 model, AML45 cells were not sensitive to S63845; however, similar to the MV411 model, CalPegA did not potentiate S63845 activity (Figure 4D).

### 2.6. CalPegA Depletes Plasma Asparagine In Vivo

**Figure 5:**
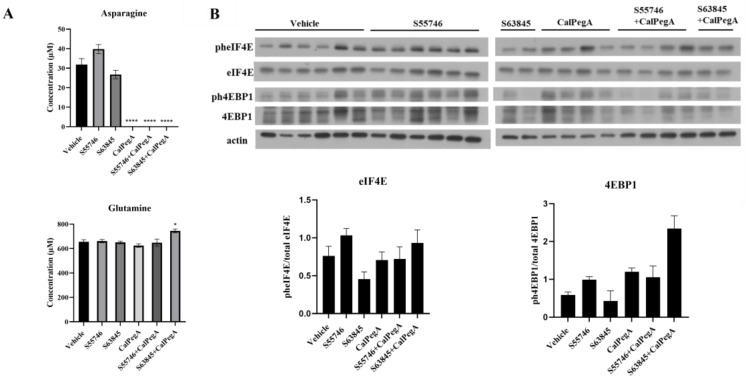
CalPegA depletes plasma asparagine in vivo but does not affect eIF4E and 4EBP1 phosphorylation status. (**A**) Plasma was isolated from whole blood of AML45-luc tumor-bearing mice and concentrations of glutamine and asparagine (µM) were measured. Statistical analyses to compare vehicle and treatment groups were performed using unpaired *t*-tests. **** *p* < 0.0001, * *p* < 0.05. (**B**) Western blot analysis of bone marrow cells isolated from AML45-luc tumor-bearing mice. Lysates were probed with the indicated antibodies, and results are expressed as the ratio of phosphorylated to total eIF4E and 4EBP1, normalized to loading control (actin). Unpaired *t*-tests were performed to compare vehicle to treatment groups and no statistically significant differences were observed.

To examine the pharmacodynamic effects of CalPegA and S55746/S63845 treatment, amino acid levels were measured in plasma collected from AML45-luc tumor-bearing mice at study termination. Asparagine concentration was not impacted in the S55746 or S63845 single-agent treatment groups but was undetectable in all CalPegA-treated groups. Although asparaginases also have glutaminase activity, CalPegA did not reduce plasma glutamine in this model (Figure 5A).

To determine whether the CalPegA-S55746 combination impacted mRNA translation initiation in vivo, we used immunoblotting to assess the phosphorylation status of eIF4E and 4EBP1 in bone marrow cells from AML45-luc tumor-bearing mice. Reduced phosphorylation of both proteins would indicate increased binding and inhibition of mRNA translation. In contrast to our in vitro findings, there was no significant change in the phosphorylation status of either eFI4E or 4EBP1 in any treatment group compared to vehicle-treated mice (Figure 5B). This observation may be related to the inability of CalPegA to deplete glutamine in vivo, as we have previously observed decreased phosphorylation of these proteins in vivo with PegC-mediated glutamine depletion [4].

## 3. Discussion

Over the past three decades, the treatment paradigm for AML has primarily relied on chemotherapy, either as a standalone approach or in combination with stem cell transplantation. Since its regulatory approval in 2018, Ven has become a cornerstone in AML therapy, demonstrating high response rates and durable remissions when combined with hypomethylating agents or low-dose cytarabine [18]. While BCL-2 inhibitors have demonstrated clinical efficacy, resistance mechanisms, including the upregulation of the anti-apoptotic protein MCL-1, remain a significant limitation. Preclinical studies indicate that co-targeting BCL-2 and MCL-1 with small molecules is highly synergistic and effective in suppressing TP53-defective AML both in vitro and in vivo [19]. Despite this promise, the clinical development of MCL-1 inhibitors has been impeded by on-target cardiotoxicity, attributable to the critical role of MCL-1 in cardiomyocyte survival [20,21]. Resistance to Ven-based regimens continues to present a therapeutic challenge, highlighting the urgent need for novel drug combinations capable of overcoming resistance or providing effective salvage options.

We previously found that the anti-leukemic activity of Ven synergized with the *Erwinia chrysanthemi* asparaginase, PegC, resulting in remarkable in vivo activity. Since there are no FDA-approved long-acting *Erwinia*-derived asparaginases, we investigated the combination of a long-acting *E. coli* asparaginase with BCL-2 inhibition. CalPegA is approved as part of a multi-agent chemotherapeutic regimen for ALL in pediatric and young adult patients [22]. Compared to pegaspargase, another clinically approved long-acting pegylated asparaginase, CalPegA is a more stable formulation with a longer half-life [23]. We hypothesized that similar to our findings with PegC, CalPegA would enhance the anti-leukemic activity of the BCL-2 inhibitor, S55746. Indeed, we report that CalPegA potentiated the anti-leukemic activity of S55746 in AML cell lines and primary patient samples in vitro as well as in orthotopic AML mouse models.

In vitro, the CalPegA + S55746 combination resulted in decreased protein synthesis and increased interaction between eIF4E and 4EBP1, suggesting impairment of cap-dependent translation, which is consistent with our findings for Ven-PegC [5]. This finding was not recapitulated in vivo, which could be related to the lack of CalPegA-mediated glutamine depletion. Although it is known that *E. coli*-derived asparaginases have less inherent glutaminase activity than crisantaspases, it was unexpected that CalPegA did not deplete glutamine. Blood for amino acid analysis was collected when CalPegA was at its trough concentration, so it is possible that glutamine was initially reduced but rebounded. Future studies should examine plasma amino acid levels at additional time points to provide a more comprehensive examination of glutamine changes. Our previous work and other reports have demonstrated that AML cells are sensitive to glutamine withdrawal and interference with glutamine metabolism [24,25], and that targeting glutaminolysis synergizes with BCL-2 inhibition [26]. However, our plasma amino acid data suggests that CalPegA-mediated asparagine depletion alone is sufficient for synergy with BCL-2 inhibition. Additional work is required to establish the contribution of glutamine versus asparagine depletion to the anti-AML activity of CalPegA. Potential future studies could directly compare the combination of S55746 with *E.coli* or *Erwinia*-derived asparaginase, or with other agents that target glutamine metabolism, such as glutaminase inhibitors or glutamine antagonists. Interestingly, CalPegA was less tolerable than PegC [5]. While some reports have suggested that the glutaminase activity of asparaginases is responsible for its toxicity, our findings provide contrary evidence, given that PegC was more tolerable despite having a higher glutaminase activity [27,28,29]. Although we cannot discount that the increased in vivo stability of CalPegA could be related to its toxicity, these results highlight the gaps of knowledge in the relationship between the glutaminase activity of asparaginases and their respective toxicity.

Our study also aimed to determine if CalPegA could potentiate the effect of MCL-1 inhibition. We found that while all human AML cell lines and primary samples tested were sensitive to both S63845 and CalPegA as single agents, the combination of the two drugs did not drastically improve anti-leukemic activity. Asparaginases have been shown to suppress mTOR pathway activation, leading to reduced levels of phosphorylated 4EBP1 and a decrease in cap-dependent translation [24], and our previous work demonstrated that PegC treatment resulted in inhibition of cap-dependent translation of proteins, including MCL-1 in AML. It is therefore plausible that CalPegA and S63845 do not synergize due to redundancy in their anti-leukemia mechanisms, but to better understand the limitations in synergy between CalPegA and MCL-1 inhibition, further mechanistic studies are required.

An interesting observation from our in vivo studies was the difference in efficacy of S63845 between the AML45 and MV411 models. S63845 as a single agent completely controlled leukemia growth in the MV411 model, whereas it had no significant impact in the AML45 model. This variability underscores the challenge posed by the heterogeneity of AML, which may impact the generalizability of the findings. While both BCL-2 and MCL-1 appear to play prominent pro-survival roles in AML, the level of reliance on either protein varies between subtypes [30]. From our studies, we found that the only AML primary sample that was negative for FTL3-ITD was the least sensitive to S557746 and S55746 activity was not potentiated by CalPegA. However, among the two cell lines without FLT3-ITD, HL60 had an S55746 IC_50_ of 0.15 ± 0.06 µM, whereas MonoMac6 cells were the least sensitive, with an IC_50_ of 6.7 ± 1.0 µM, so the response is likely influenced by the presence of additional mutations. In contrast to published reports that the presence of mutations in IDH1 or IDH2 is correlated with response to Ven, the primary patient sample with the highest S55746 IC_50_ had an IDH1 mutation (AML32). Future studies should be expanded to include comprehensive genomic analyses with additional primary patient samples to identify biomarkers that predict response to BCL-2 and MCL-1 inhibitors, as well as their combination with CalPegA.

Overall, our work demonstrates that the long-acting *E. coli* asparaginase CalPegA enhances the anti-leukemic effect of the BCL-2 inhibitor S55746, but does not impact the activity of the MCL-1 inhibitor S63845 in AML cell lines, patient-derived primary AML samples, and in AML xenograft mouse models. This work supports the continued clinical development of S55746 in combination with CalPegA as a novel therapeutic regimen in AML.

## 4. Materials and Methods

### 4.1. Cell Culture and Reagents

The human AML cell lines MOLM-14, MonoMac6, MV4-11, and HL60 were provided by the Translational Lab Shared Service at the University of Maryland Baltimore. HL60 cells were cultured in IMDM (Life Technologies, Carlsbad, CA, USA) supplemented with 10% fetal bovine serum (FBS) (GeminiBio, West Sacramento, CA, USA). All other cell lines were cultured in RPMI-1640 (Life Technologies) supplemented with 10% FBS. Primary human AML cells were obtained through an IRB-approved institutional tissue procurement protocol at the University of Maryland Baltimore and processed and cultured as previously described [5]. CalPegA, S55746, and S63845 were provided by Servier Pharmaceuticals (Boston, MA, USA).

### 4.2. Cell Proliferation Assay

Cells were seeded into 96-well plates, and approximately 18 h later, cells were treated with serially diluted CalPegA, S55746, and S63845. For drug combination assays, cells were treated with serially diluted S63845 or S55746 alone or with a low dose (IC_20_) of CalPegA. After 72 h, water-soluble tetrazolium (WST-1) (Clontech, Mountain View, CA, USA) was added, and plates were read using a BioTek Synergy H1 plate reader (BioTek, Winooski, VT, USA) after 4 additional hours of incubation at 37 °C. For primary AML cells, assays were terminated 48 h after treatment using alamarBlue reagent. Data were analyzed and graphed using GraphPad Prism Version 8.1.2 (GraphPad, La Jolla, CA, USA), and IC_50_ concentrations were calculated.

### 4.3. Cell Death/Culture Expansion Assays

Cells were seeded into 6-well cell culture plates and treated the following day with either vehicle or CalPegA, S55746, or S63845 alone and in combination at the IC_50_ values. The percentage of viable cells was measured by trypan blue exclusion.

### 4.4. Combination Synergy Assay

For determining synergism, cell lines were seeded into 96-well plates and treated the next day with serially-diluted CalPegA, S55746, and S63845 alone and in combination at fixed ratios. Cultures were terminated 72 h after treatment with WST-1. After 4 h of additional incubation, plates were read at 37 °C using a BioTek Synergy H1 plate reader. Data were analyzed by median effect analysis using Compusyn software (free online software based on the Chou Talalay theorem). A combination index (CI) was generated; CI < 1 synergistic, CI = 1 additive, and CI > 1 antagonistic.

### 4.5. Western Blot Analysis

Cells were lysed with radioimmunoprecipitation assay (RIPA) buffer (Millipore, Burlington, MA, USA) supplemented with protease and phosphatase inhibitor cocktails (Sigma Aldrich, St. Louis, MI, USA). Lysates were incubated on ice for 10 min and then centrifuged at 14,000× *g* at 4 °C for 15 min. The protein content of lysates was determined using Bradford Dye Reagent (BioRad, Hercules, CA, USA), and lysates were separated by 4–15% polyacrylamide gels (BioRad) and then transferred onto polyvinylidene difluoride (PVDF) membranes (BioRad). Membranes were blocked with either 5% non-fat milk or bovine serum albumin in tris-buffered saline with 0.1% Tween 20 (TBST), incubated with primary antibodies at 4 °C overnight, and then incubated with HRP-conjugated secondary antibody for 1 h. Bands were visualized using Clarity Western Enhanced Chemiluminescence (ECL) substrate (BioRad) or SuperSignal West Pico Plus ECL substrate (Thermo Fisher Scientific). Densitometric analyses were performed using ImageJ Version 1.52a (NIH).

Antibodies against actin, GAPDH, phosphorylated/total 4EBP1 and eIF4E, and horseradish peroxidase (HRP)-conjugated secondary antibodies were purchased from Cell Signaling Technology (Danvers, MA, USA).

### 4.6. Puromycin Incorporation Assay (SUnSET Assay)

The SUnSET (surface sensing of translation) assay was performed per the manufacturer’s recommendations (Kerafast, Boston, MA, USA). Briefly, cells were treated with either vehicle or CalPegA, S55746, or S63845 alone or in combination at the IC_50_ values for 24 h and then incubated for 20 min at 37 °C with 1 µg/mL puromycin (Sigma Aldrich). Cells were washed with PBS used for SDS-PAGE/immunoblotting. Antibody against puromycin was purchased from Sigma Aldrich.

### 4.7. Immunoprecipitation

Cells were seeded into T25 cell culture flasks and treated the next day with either vehicle, CalPegA, S55746, or S63845 alone or in combination at the IC_50_ values. After 24 h, the cells were lysed, and 250 µg of protein for each lysate was precleared with Protein A beads for 1 h at 4 °C on a rotator. Lysates were then incubated with 4EBP1 antibody at 4 °C overnight, followed by incubation with Protein A beads for 2 h at 4 °C. Beads were washed 5 times in RIPA buffer, resuspended in 2X SDS sample loading buffer, and boiled at 95 °C for 5 min, and then separated on 4–15% polyacrylamide gels.

### 4.8. In Vivo Mouse Studies

All experiments were conducted in compliance with Public Health Service (PHS) guidelines for animal research and approved by the University of Maryland Baltimore Institutional Animal Care and Use Committee. To determine the tolerability of S55746, 63845, and CalPegA, 6-to-8-week-old NRG mice (3 mice/group) were treated with 3 doses (low, medium, or high) of each drug, and weight was monitored 5 days a week. S55746 was given at 25, 50, and 100 mg/kg by oral gavage 5 days a week (Monday–Friday). S63845 was given IV at 25 mg/kg twice a week (Mon and Thurs), 50 mg/kg once a week, and 50 mg/kg twice weekly. CalPegA was given IV once a week at 250, 500, and 1000 IU/kg.

For the MV411 orthotopic model, 1 × 10^6^ luciferase-expressing MV411 cells were injected IV into NRG mice. Leukemia engraftment was confirmed using the Xenogen IVIS-200 system (Alameda, CA, USA), and mice were sorted into groups with equal mean leukemia burden. Mice were treated with either vehicle, S55746 (orally, 75 mg/kg, 5 days per week), S63845 (IV, 25 mg/kg, 2 days a week), CalPegA (IV, 100 IU/kg, once weekly), or the combination of CalPegA + S55746 or CalPegA + S63845 starting on the day of sorting. Mice were imaged to measure leukemia burden weekly, and body weight was monitored.

The AML45-luc model was developed as previously described [4]. AML45-luc cells (1 ×  10^6^) were injected IV into NRG mice. Mice were imaged 3–10 days later using the Xenogen IVIS-200 System (Alameda, CA, USA) and sorted into groups with equal mean leukemia burden. Dosing began on the day of sorting with dosages the same as described for the MV411 model. Mice were imaged to measure leukemia burden weekly, and body weight was monitored. After day 36, the CalPegA dose was decreased to 75 IU/kg after weight loss was observed in CalPegA-treated mice. On day 78 post-start of dosing, all study mice were euthanized.

### 4.9. Plasma Amino Acid Measurement

Plasma was isolated from whole blood and frozen at −20 °C until analysis. Free AA concentrations were measured using a Biochrom 30 or Biochrom 30+ Amino Acid Analyzer (Biochrom Ltd., Cambridge, UK) by cation-exchange chromatography and ninhydrin detection according to the manufacturer’s instructions. Results were quantified using commercially available calibration standards and normalized to the internal standard s-2-aminoethylcysteine and reported as μmol/L (µM). Standard curves were generated for each AA, and AAs were quantified. Two quality control standards were evaluated as an unknown at the beginning of each set of sample runs.

### 4.10. Statistics

Data are expressed as means ± standard error of the mean (SEM). Unpaired *t*-tests were performed to compare the effects of individual treatment groups to the vehicle treatment group, and analysis of variance (ANOVA) followed by Bonferroni’s post hoc correction was used to compare differences among multiple groups. Statistical analyses were performed using GraphPad Prism Version 8.1.2 (La Jolla, CA, USA).

## Data Availability

The original contributions presented in the study are included in the article/Supplementary Materials, further inquiries can be directed to the corresponding author/s.

## Supplementary Materials

The following supporting information can be downloaded at: https://www.mdpi.com/article/10.3390/ijms252313091/s1.
