# Supplementary material for: Calaspargase-Pegol-Mknl Combined with BCL-2 and MCL-1 Inhibition for Acute Myeloid Leukemia
Source: Int J Mol Sci. 2024 Dec 5;25(23):13091. doi: 10.3390/ijms252313091 (PMC11641566; doi:10.3390/ijms252313091)
Supplement: Supplementary file 1 [file ijms-25-13091-s001.zip › ijms-3227838-supplementary.pdf]

Supplementary Figures

AML612: Karyotype: 46,XX

- *FLT3*-ITD (ITD variant 411 base pair with an insertion length of 83 bp and allelic burden of 49%)
- *DNMT3A* (R882P) with VAF 44%
- *NPM1* (W288fs) with VAF 28%

AML615: Karyotype: 46,XX,t(5;17)(q35;q21)

- *FLT3*-ITD (ITD variant 391 base pair with an insertion length of 63 bp and allelic burden of 68%)

AML673: Karyotype: 46,XX,t(6;9)(p22;q34) – Gene Fusion: DEK-NUP214

- *FLT3*-ITD (ITD variant 405 base pair with an insertion length of 77 bp and allelic burden of 111%)
- NRAS (G13V) with VAF 49%

AML32: Karyotype: 46,XX,[20]

- IDH1 (R132)
- NRAS (G13D)
- DNMT3A (R882C)
- NPMI (W288fs)

Supplementary Figure S1: Cytogenetic and molecular mutation information for primary AML patient samples

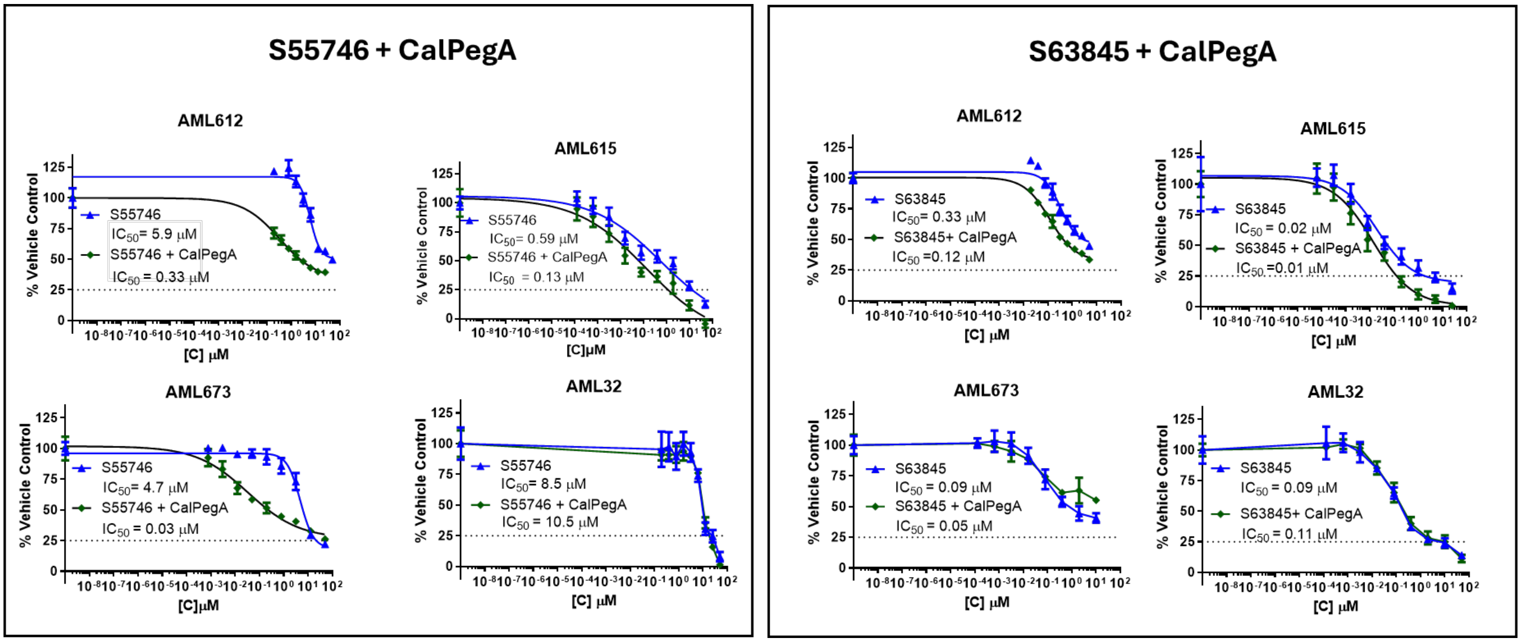

Supplementary Figure S2: S55746 is enhanced by CalPegA in primary AML patient samples. Primary AML cells were plated overnight then treated the next day with serially diluted S55746 or S63845 alone or in combination with a low dose (IC_{20}) of CalPegA. Cell proliferation was assessed using alamarBlue 48h after treatment.

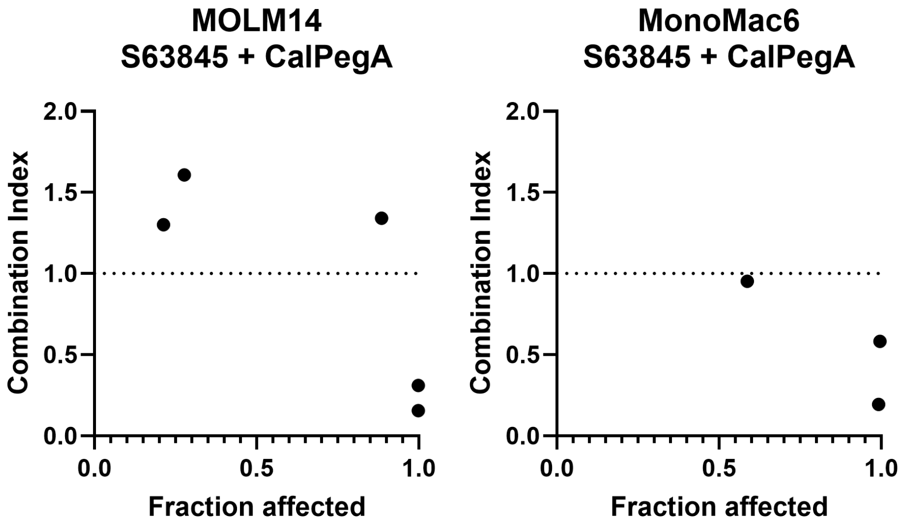

Supplementary Figure S3: S63845 and CalPegA synergy. MOLM14 and MonoMac6 cells were treated with fixed ratio doses of CalPegA and S63845 for 72 h followed by WST-1 termination. Combination indexes (CI) were calculated using Compusyn Software.

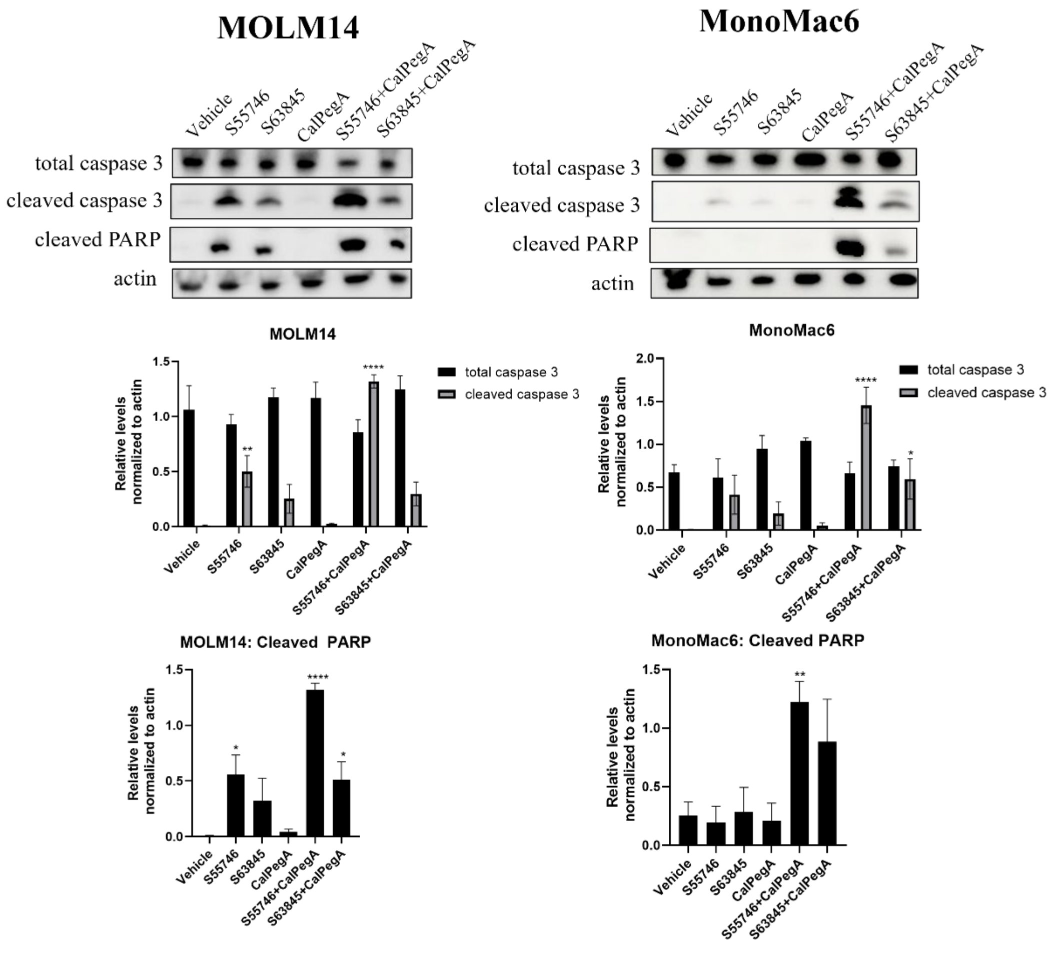

Supplementary Figure S4: Induction of apoptosis. MOLM14 and MonoMac6 cells were treated with vehicle or S55746, S63845, or CalPegA alone and in combination at the IC_{50} values for 24h. After 24h, cells were harvested and immunoblotting of whole cell protein extracts was performed using the indicated antibodies. Statistical analyses to compare vehicle to treatment groups were performed using ANOVA. The bar diagram represents densitometric quantification of three independent experiments normalized to the vehicle control. ****$p<0.0001$,**$p < 0.01$, *$p < 0.05$.

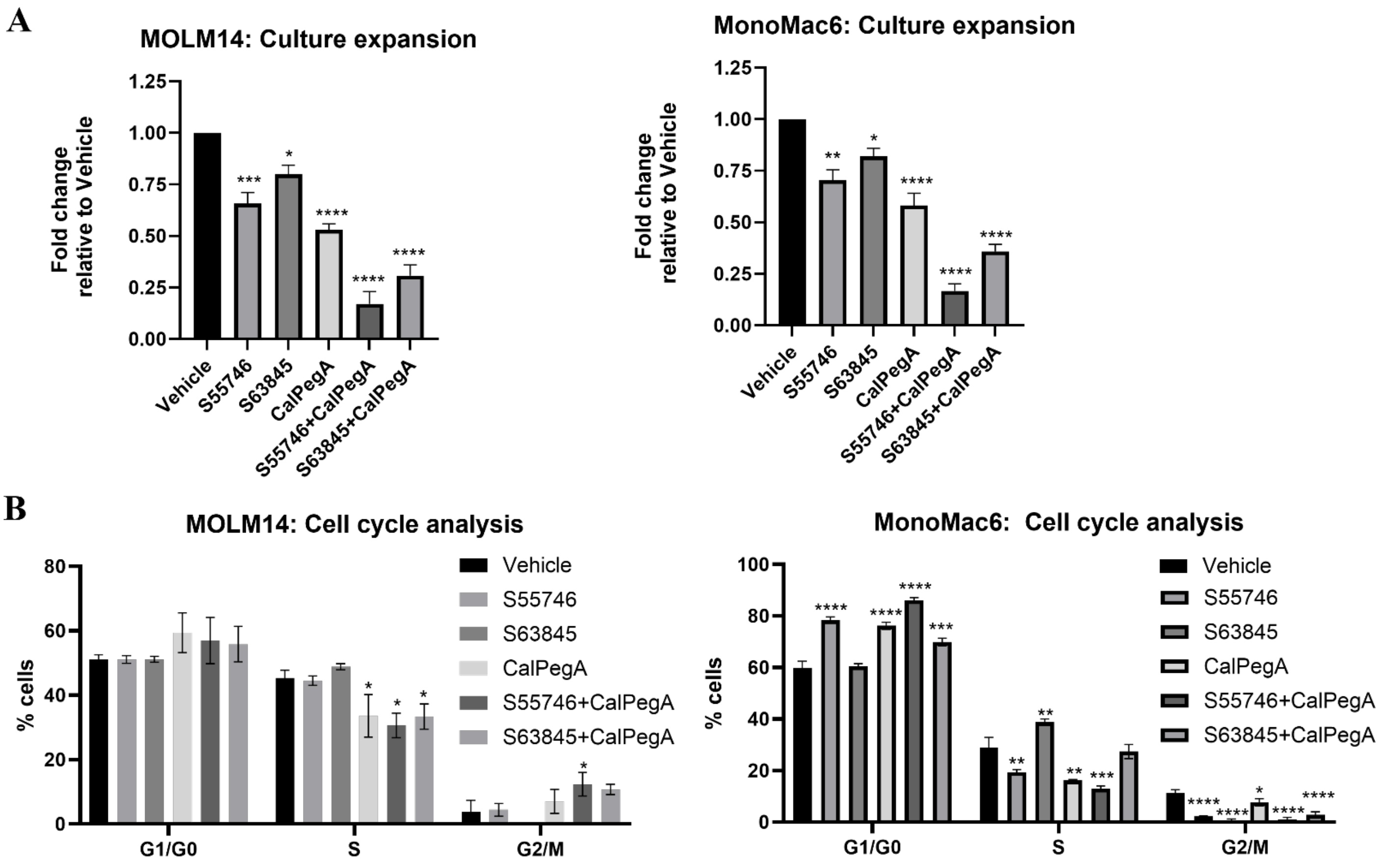

Supplementary Figure S5: Culture expansion and cell cycle analysis. (A) MOLM14 and MonoMac6 cells were treated with vehicle or S55746, S63845, or CalPegA alone and in combination at the IC_{50} values for 72h. After 72 h, cell culture expansion was calculated by dividing the total number of viable cells by the number of cells initially plated. Data are expressed as fold change normalized to vehicle control (fold change for each treatment group was divided by the fold change for the vehicle-treated cells). Statistical analyses to compare vehicle to treatment groups were performed using ANOVA. ****p<0.0001, ***p<0.001 ,**p < 0.01, *p < 0.05. **(B)** MOLM14 and MonoMac6 cells were treated with either vehicle or S55746, S63845, or CalPegA alone and in combination at the IC_{50} values. After 72h, harvested cells were permeabilized and fixed and stained with propidium iodide to measure total DNA content. Cells were analyzed by flow cytometry and the percentage of cells in each cell cycle phase was calculated using FCS Express Version 7. Statistical analyses to compare vehicle to treatment groups were performed using ANOVA. ****p<0.0001, ***p<0.001 ,**p < 0.01, *p < 0.05.

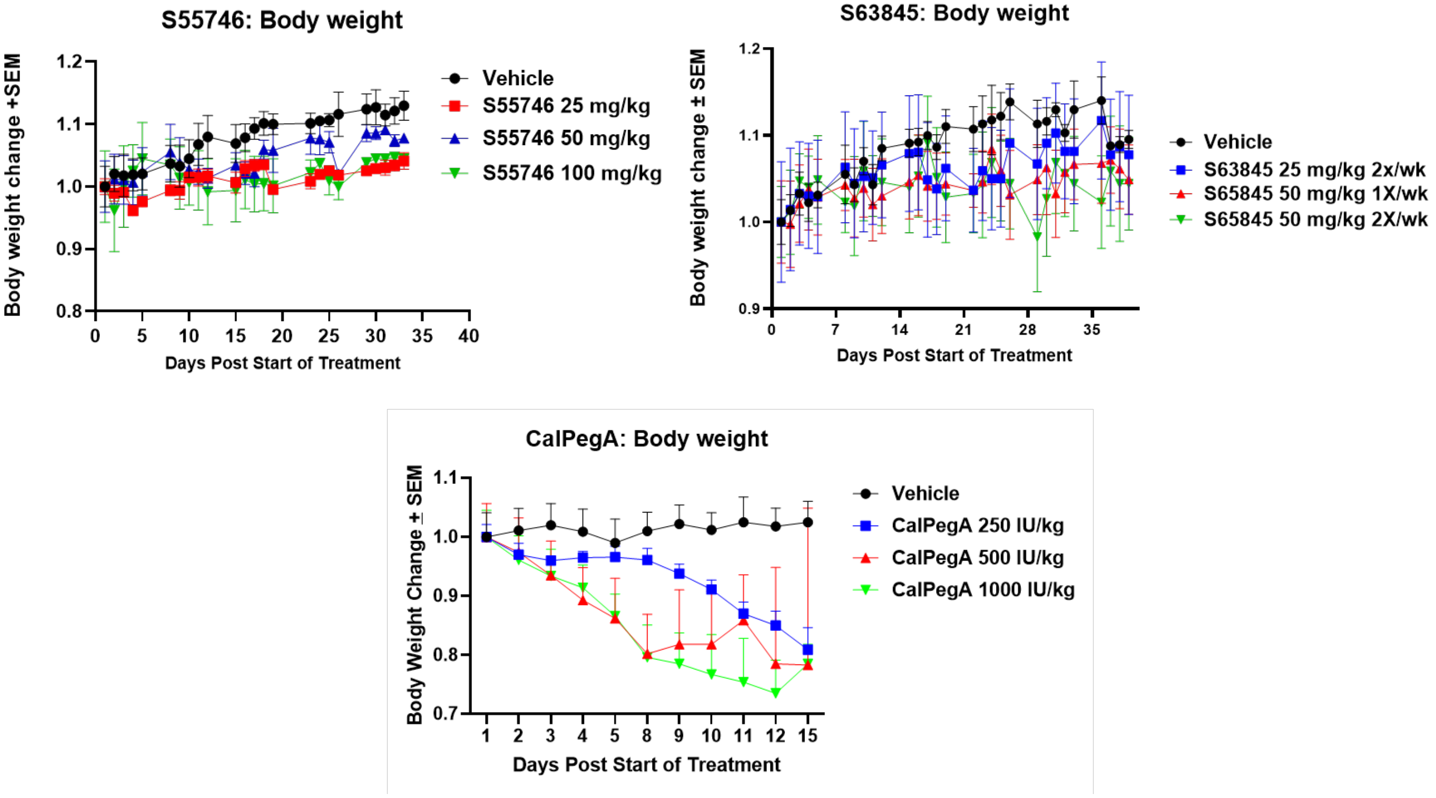

Supplementary Figure S6: Tolerability of S55746, S63845, and CalPegA. Healthy NRG mice treated with vehicle control or increasing doses of each agent at the indicated concentrations. Body weight over time relative to vehicle-treated mice was calculated as an indicator of toxicity.
